# Magnesium neuroprotection in retinal ganglion cells: A computational study of frequency-dependent therapeutic windows and intervention timing

**DOI:** 10.1371/journal.pone.0348068

**Published:** 2026-06-01

**Authors:** Mehdi Borjkhani, Hadi Borjkhani, Morteza A. Sharif

**Affiliations:** 1 International Centre for Translational Eye Research (ICTER), Institute of Physical Chemistry, Polish Academy of Sciences, Warsaw, Poland; 2 Institute of Physical Chemistry, Polish Academy of Sciences, Warsaw, ‌‌Poland; 3 Faculty 1, University of Applied Sciences (HTW) Berlin, Berlin, Germany; 4 Optics and Laser Engineering Group, Faculty of Electrical Engineering,‌‌ Urmia University of Technology, Urmia, Iran; Georgia State University, UNITED STATES OF AMERICA

## Abstract

Retinal ganglion cells (RGCs) are vulnerable to excitotoxic damage mediated by excessive NMDA receptor activation and calcium overload. Extracellular magnesium (Mg^2+^) blocks NMDA receptors in a voltage-dependent manner, offering potential neuroprotection. However, the optimal Mg^2+^ concentrations and timing for effective intervention remain poorly defined. We developed a conductance-based computational model of an RGC incorporating Hodgkin-Huxley dynamics, AMPA and NMDA receptor-mediated synaptic transmission, and intracellular calcium dynamics. We systematically varied Mg^2+^ concentration (0.2–2.5 mM) and stimulation frequency (10–100 Hz) to identify therapeutic windows balancing neuroprotection with function preservation. At physiological frequencies (10–60 Hz), elevated Mg^2+^ reduced calcium (Ca^2+^) accumulation by 50–85% without affecting spike output. At excitotoxic frequencies (80 Hz), a narrow therapeutic window of 1.6–2.0 mM was identified, lying within a broader 1.4–2.0 mM spike-loss plateau (20% loss), where calcium additionally fell below the toxicity threshold while spike output was preserved. Intervention timing analysis revealed that Mg^2+^ protection efficacy is maximal with pre-treatment or immediate intervention (100%), and declines steeply with delay—reflecting the rapid early rise in Ca^2+^ rather than a fixed biological deadline (≥50% protection requires intervention within 0.2 s in our abrupt-onset protocol; ∼11% by 0.5 s). Re-analysis in terms of normalized Ca^2+^ progress revealed that the critical constraint for ≥50% protection is intervention before ∼35% of peak Ca^2+^ accumulation—a state-based threshold reflecting relative phase sensitivity that generalizes across timescales. Sensitivity analyses confirmed robustness of the therapeutic window across physiologically plausible parameter ranges, and numerical validation demonstrated accuracy of the computational approach. These findings demonstrate that Mg^2+^-mediated neuroprotection is highly dependent on both concentration and timing, with implications for therapeutic strategies targeting glutamate excitotoxicity in glaucoma and retinal ischemia.

## Introduction

Retinal ganglion cells (RGCs) are the sole output neurons of the retina, transmitting visual information from the eye to the brain via their axons, which form the optic nerve [1]. These neurons are particularly vulnerable to excitotoxic damage, a pathological process in which excessive glutamatergic signaling leads to neuronal death [2,3]. RGC loss is the defining feature of glaucoma, the leading cause of irreversible blindness worldwide, affecting over 70 million people [4,5]. Recent advances in understanding RGC death mechanisms, including caspase-mediated apoptotic pathways and calcium homeostasis disruption, have identified multiple potential therapeutic targets [6,7]. Additionally, RGCs are damaged in other ophthalmic conditions including retinal ischemia, diabetic retinopathy, and traumatic optic neuropathy [3,8]. Despite significant advances in understanding RGC pathophysiology, effective neuroprotective therapies remain elusive, highlighting the urgent need for novel therapeutic strategies [9].

### Glutamate excitotoxicity in retinal ganglion cells

Glutamate is the primary excitatory neurotransmitter in the retina, mediating synaptic transmission from bipolar cells to RGCs [1,10]. Under physiological conditions, glutamate release is tightly regulated, and RGCs respond appropriately to visual stimuli through activation of ionotropic glutamate receptors, including α-amino-3-hydroxy-5-methyl-4-isoxazolepropionic acid (AMPA) and N-methyl-D-aspartate (NMDA) receptors [11]. However, pathological conditions such as ischemia, elevated intraocular pressure, or metabolic stress can trigger excessive glutamate release and impaired reuptake, leading to sustained receptor activation [2,3].

NMDA receptors play a central role in excitotoxicity due to their high permeability to calcium ions (Ca^2+^) [10,12]. Unlike AMPA receptors, which primarily conduct sodium and potassium, NMDA receptors allow substantial Ca^2+^ influx upon activation [13]. While physiological Ca^2+^ signaling is essential for synaptic plasticity and normal cellular function, excessive Ca^2+^ accumulation activates destructive enzymatic cascades, including calpains, phospholipases, and endonucleases, ultimately leading to cell death [10,14]. This calcium-mediated excitotoxicity has been implicated in numerous neurodegenerative conditions, making NMDA receptor modulation an attractive therapeutic target [10,15].

### Magnesium as an endogenous NMDA receptor modulator

A unique property of NMDA receptors is their voltage-dependent block by extracellular magnesium ions (Mg^2+^) [16,17]. At resting membrane potentials, Mg^2+^ ions occupy the receptor channel pore, preventing ion flux despite glutamate binding. Membrane depolarization relieves this block, allowing current flow through the receptor [18]. Recent cryo-electron microscopy (cryo-EM) structural studies have identified three distinct Mg^2+^-binding sites on NMDA receptors, revealing that Mg^2+^ exerts multifaceted regulatory effects beyond simple pore block, including allosteric modulation through the N-terminal domain [19]. This voltage-dependent gating mechanism underlies the role of NMDA receptors as coincidence detectors, requiring both presynaptic glutamate release and postsynaptic depolarization for activation [11,20].

The Mg^2+^ block of NMDA receptors has attracted considerable interest as a potential neuroprotective mechanism [10,21]. Elevated extracellular Mg^2+^ enhances the voltage-dependent block, reducing Ca^2+^ influx through NMDA receptors even during pathological glutamate exposure [18]. Importantly, because the block is voltage-dependent rather than absolute, Mg^2+^ supplementation may provide neuroprotection while partially preserving NMDA receptor function, potentially avoiding the adverse effects associated with complete receptor antagonism [10,12].

Clinical and experimental evidence supports the neuroprotective potential of Mg^2+^. In stroke models, Mg^2+^ administration reduced infarct volume and improved functional outcomes [22]. The Field Administration of Stroke Therapy–Magnesium (FAST-MAG) trial demonstrated the feasibility of prehospital Mg^2+^ therapy in acute stroke patients, though the trial showed no significant overall functional benefit; post-hoc analyses suggested possible efficacy in specific stroke subtypes [23]. In the visual system, Mg^2+^ supplementation has shown promise in animal models of glaucoma and retinal ischemia [3,10]. However, clinical translation has been hampered by incomplete understanding of the optimal Mg^2+^ concentrations and timing required for effective neuroprotection without compromising neural function.

### The therapeutic window challenge

A critical challenge in Mg^2+^-based neuroprotection is identifying the therapeutic window—the range of concentrations that provide meaningful protection against excitotoxicity while preserving normal synaptic transmission [10]. Too little Mg^2+^ fails to adequately block NMDA receptors during pathological activation, while excessive Mg^2+^ may impair physiological signaling. Physiological extracellular Mg^2+^ concentration in cerebrospinal fluid is approximately 0.8–1.0 mM [24], and therapeutic strategies typically aim for mild hypermagnesemia (1.5–2.5 mM) to enhance neuroprotection [22].

Complicating this picture is the frequency-dependence of NMDA receptor contribution to synaptic transmission. At low stimulation frequencies, AMPA receptors dominate fast synaptic transmission, and NMDA receptor block has minimal functional impact [11]. However, at high frequencies characteristic of excitotoxic conditions, NMDA receptors contribute significantly to temporal summation and sustained depolarization [25]. This frequency-dependence suggests that the therapeutic window may vary with the level of synaptic activity, a hypothesis that has not been systematically investigated.

Additionally, the timing of Mg^2+^ intervention relative to excitotoxic insult onset may critically influence efficacy. Calcium accumulation begins rapidly upon excessive NMDA receptor activation, and delayed intervention may fail to prevent irreversible damage [14]. Understanding the temporal constraints on Mg^2+^ neuroprotection is essential for clinical translation, particularly in acute conditions such as retinal ischemia where treatment delays are common.

### Computational modeling of RGC neuroprotection

Computational modeling provides a powerful approach to systematically investigate the complex parameter space of Mg^2+^ neuroprotection [26]. Conductance-based models incorporating Hodgkin-Huxley-type dynamics [27] can capture the voltage-dependent interactions between intrinsic conductances, synaptic receptors, and Mg^2+^ block with high temporal resolution. Such models enable exploration of conditions that would be difficult or impossible to study experimentally, including systematic variation of Mg^2+^ concentration, stimulation frequency, and intervention timing.

Previous computational studies have examined various aspects of RGC physiology, including intrinsic firing properties [28], responses to electrical stimulation [29], and synaptic integration [30]. Computational approaches have also proven valuable for understanding NMDA and AMPA receptor dynamics in the context of synaptic plasticity and pathological conditions [12,31]. However, comprehensive modeling of Mg^2+^-mediated neuroprotection in RGCs, particularly addressing the frequency-dependence of therapeutic windows and temporal constraints on intervention, has not been reported.

### Aims and overview of the present study

In this study, we developed a biophysically detailed computational model of an RGC to investigate the neuroprotective effects of extracellular Mg^2+^ during glutamatergic excitotoxicity. Our model incorporates voltage-gated sodium, potassium, and calcium conductances; AMPA and NMDA receptor-mediated synaptic currents; the voltage-dependent Mg^2+^ block of NMDA receptors following the Jahr-Stevens formulation [18]; and intracellular calcium dynamics.

We systematically varied extracellular Mg^2+^ concentration (0.2–2.5 mM) and glutamatergic stimulation frequency (10–100 Hz) to address three key questions: (1) What are the frequency-dependent therapeutic windows for Mg^2+^ neuroprotection? (2) How does the trade-off between neuroprotection and function preservation vary with activity level? (3) What are the temporal constraints on Mg^2+^ intervention during excitotoxic stress?

Our results reveal a narrow but clinically relevant therapeutic window at excitotoxic frequencies, identify a spike loss plateau phenomenon that creates an optimal zone for neuroprotection, and demonstrate that Mg^2+^ protection efficacy declines steeply with intervention delay.

## Materials and methods

### Computational model

We developed a single-compartment conductance-based model of an RGC to investigate the neuroprotective effects of Mg^2+^ during glutamatergic excitotoxicity. The model incorporates Hodgkin-Huxley-type intrinsic conductances [27], glutamatergic synaptic inputs via AMPA and NMDA receptors, and intracellular calcium dynamics. The model architecture was informed by previous computational studies of RGC electrophysiology [28,29].

### Rationale for single-compartment approach

The single-compartment approach was chosen based on the relatively compact dendritic morphology of RGCs, where the soma is electrotonically close to synaptic input sites, making somatic recordings representative of integrated synaptic responses [28]. Unlike pyramidal neurons with extensive dendritic arbors (1000+ μm), RGC dendritic trees typically span 100–300 μm, with relatively uniform electrotonic properties. This approach enables systematic exploration of the fundamental Mg^2+^ concentration–function trade-off while maintaining computational tractability and interpretability. The implications of dendritic compartmentalization are addressed in the Discussion.

### Membrane dynamics

The membrane potential *V* evolves according to:


$$\begin{array}{c} C_{m}\frac{dV}{dt}=-I_{\text{Na}}-I_{\text{Kdr}}-I_{\text{KA}}-I_{\text{CaL}}-I_{\text{KCa}}-I_{L}-I_{\text{AMPA}}-I_{\text{NMDA}} \end{array}$$ (1)

where $C_{m}=1.0\;\mu {\text{F/cm}}^{2}$ is the membrane capacitance [32]. The ionic currents include voltage-gated sodium (*I*_Na_), delayed rectifier potassium (*I*_Kdr_), A-type potassium (*I*_KA_), L-type calcium (*I*_CaL_), calcium-activated potassium (*I*_KCa_), and leak (*I*_*L*_) conductances.

### Intrinsic conductances

Voltage-gated currents followed standard Hodgkin-Huxley formalism [27]:


$$\begin{array}{c} I_{\text{Na}}=g_{\text{Na}}m^{3}h(V-E_{\text{Na}}) \end{array}$$ (2)

$$\begin{array}{c} I_{\text{Kdr}}=g_{\text{Kdr}}n^{4}(V-E_{K}) \end{array}$$ (3)

$$\begin{array}{c} I_{\text{KA}}=g_{\text{KA}}a^{3}b(V-E_{K}) \end{array}$$ (4)

$$\begin{array}{c} I_{\text{CaL}}=g_{\text{CaL}}s^{2}(V-E_{\text{Ca}}) \end{array}$$ (5)

$$\begin{array}{c} I_{L}=g_{L}(V-E_{L}) \end{array}$$ (6)

The A-type potassium current kinetics were based on Connor and Stevens [33], while L-type calcium channel properties followed Fox et al. [34]. The calcium-activated potassium current was modeled as:


$$\begin{array}{c} I_{\text{KCa}}=g_{\text{KCa}}\cdot \frac{[{\text{Ca}}^{2+}{]}_{i}^{2}}{[{\text{Ca}}^{2+}{]}_{i}^{2}+K_{d}^{2}}\cdot (V-E_{K}) \end{array}$$ (7)

with $K_{d}=0.5\;\mu$M [35].

Conductance values were: *g*_Na_ = 120 mS/cm^2^, *g*_Kdr_ = 36 mS/cm^2^, *g*_KA_ = 8 mS/cm^2^, *g*_CaL_ = 0.3 mS/cm^2^, *g*_KCa_ = 0.3 mS/cm^2^, and $g_{L}=0.35$ mS/cm^2^. Reversal potentials were: *E*_Na_ = +50 mV, $E_{K}=-77$ mV, *E*_Ca_ = +120 mV, $E_{L}=-54.4$ mV, and resting potential $V_{\text{rest}}=-65$ mV, consistent with experimental measurements in mammalian RGCs [36,37]. Complete parameter values including synaptic kinetics are provided in Tables 1,2 and S2 Table.

**Table 1:** Model parameters: Intrinsic properties. Membrane properties, reversal potentials, and ionic conductances. Complete parameters with full references are provided in S2 Table.

Parameter	Value	Unit	Parameter	Value	Unit
*C*_*m*_	1.0	μF/cm^2^	*g*_Na_	120	mS/cm^2^
*V*_rest_	−65	mV	*g*_Kdr_	36	mS/cm^2^
*E*_Na_	+50	mV	*g*_KA_	8	mS/cm^2^
*E*_K_	−77	mV	*g*_CaL_	0.3	mS/cm^2^
*E*_Ca_	+120	mV	*g*_KCa_	0.3	mS/cm^2^
*E*_*L*_	−54.4	mV	*g*_*L*_	0.35	mS/cm^2^
*E*_exc_	0	mV			

**Table 2:** Model parameters: Synaptic and Ca^2+^ dynamics. Synaptic conductances, receptor kinetics, Mg^2+^ block parameters, calcium dynamics, and stimulation protocol. Primary references: Hodgkin & Huxley 1952 [27]; Jahr & Stevens 1990 [18]; Patneau & Mayer 1990 [39]; Destexhe et al. 1994 [38]. The calcium scaling factors *k*_Ca,NMDA_ and *k*_Ca,CaL_ were fitted to experimental data (see Methods); full sourcing for all parameters is provided in S2 Table.

Parameter	Value	Unit	Parameter	Value	Unit
*g*_AMPA_	0.25	mS/cm^2^	η	0.28	mM^−1^
*g*_NMDA_	1.2	mS/cm^2^	γ	0.062	mV^−1^
EC_50,AMPA_	0.5	mM	*f*_Ca_	0.15	—
EC_50,NMDA_	2	μM	$[{\text{Ca}}^{2+}{]}_{\text{rest}}$	0.05	μM
$\tau_{\text{AMPA}}$	0.3/3	ms	$\tau_{\text{Ca}}$	200	ms
$\tau_{\text{NMDA}}$	5/80	ms	*K*_*d*_(KCa)	0.5	μM
[Glu]	1.0	mM	*t*_pulse_	2	ms

### Synaptic conductances

Glutamatergic input activated both AMPA and NMDA receptor-mediated currents [11]:


$$\begin{array}{c} I_{\text{AMPA}}=g_{\text{AMPA}}s_{\text{AMPA}}(V-E_{\text{exc}}) \end{array}$$ (8)

$$\begin{array}{c} I_{\text{NMDA}}=g_{\text{NMDA}}s_{\text{NMDA}}B(V,[{\text{Mg}}^{2+}{]}_{o})(V-E_{\text{exc}}) \end{array}$$ (9)

where *E*_exc_ = 0 mV, *g*_AMPA_ = 0.25 mS/cm^2^, and *g*_NMDA_ = 1.2 mS/cm^2^.

Synaptic gating variables *s* followed first-order kinetics with glutamate-dependent activation [31,38]:


$$\begin{array}{c} \frac{ds}{dt}=\frac{\left[\text{Glu}\right]}{[\text{Glu}]+{\text{EC}}_{50}}\cdot \frac{1-s}{\tau_{\text{rise}}}-\frac{s}{\tau_{\text{decay}}} \end{array}$$ (10)

where EC_50_ (half-maximal effective concentration) values were EC_50,AMPA_ = 0.5 mM and ${\text{EC}}_{50,\text{NMDA}}=2\;\mu$M [39], with decay time constants $\tau_{\text{AMPA,decay}}=3$ ms [40] and $\tau_{\text{NMDA,decay}}=80$ ms [41]. Rise time constants were $\tau_{\text{AMPA,rise}}=0.3$ ms and $\tau_{\text{NMDA,rise}}=5$ ms.

### Voltage-dependent Mg^2+^ block of NMDA receptors

The voltage-dependent Mg^2+^ block of NMDA receptors was modeled following Jahr and Stevens [18]:


$$\begin{array}{c} B(V,[{\text{Mg}}^{2+}{]}_{o})=\frac{1}{1+\eta [{\text{Mg}}^{2+}{]}_{o}\exp(-\gamma V)} \end{array}$$ (11)

where η=0.28 mM^-1^ and γ=0.062 mV^-1^. This function captures the relief of Mg^2+^ block at depolarized potentials, a hallmark property of NMDA receptors that underlies their role as coincidence detectors [16,17].

### Intracellular calcium dynamics

Intracellular calcium concentration evolved according to a single-compartment model [42]:


$$\begin{array}{c} \frac{d[{\text{Ca}}^{2+}{]}_{i}}{dt}=-k_{\text{Ca,NMDA}}\cdot f_{\text{Ca}}\cdot I_{\text{NMDA}}-k_{\text{Ca,CaL}}\cdot I_{\text{CaL}}-\frac{[{\text{Ca}}^{2+}{]}_{i}-[{\text{Ca}}^{2+}{]}_{\text{rest}}}{\tau_{\text{Ca}}} \end{array}$$ (12)

where *f*_Ca_ = 0.15 represents the fractional calcium current through NMDA receptors [13], *k*_Ca,NMDA_ = 0.012 and *k*_Ca,CaL_ = 0.003 are dimensionless scaling factors (fitted to produce peak Ca^2+^ transients in the 0.5–5 μM range during excitotoxic stimulation, consistent with experimental measurements in cultured neurons [42,43]; these factors implicitly absorb the conversion from current density [μA/cm^2^] to concentration [μM] given the single-compartment geometry), $[{\text{Ca}}^{2+}{]}_{\text{rest}}=0.05\;\mu$M [44], and $\tau_{\text{Ca}}=200$ ms reflects combined buffering and extrusion [42].

## Simulation protocol

### Glutamatergic stimulation

Glutamate release was simulated as brief (2 ms) pulses of 1 mM concentration, approximating synaptic cleft concentrations during vesicular release [45]. Stimulation frequencies of 10, 30, 60, 80, 90, and 100 Hz were tested, representing physiological to excitotoxic stimulation levels. *Note on frequency interpretation*: The 80 + Hz stimulation frequencies serve as modeling proxies for the sustained receptor activation that occurs during pathological glutamate accumulation (e.g., during ischemia), rather than representing directly measured physiological firing rates. This approach enables systematic parameter exploration while capturing the essential feature of excitotoxicity: prolonged, intense glutamatergic stimulation exceeding normal physiological ranges. Each simulation ran for 3000 ms total. Pulsatile glutamatergic input was applied from *t* = 0 ms; however, to minimize initialization artifacts, we excluded the first 500 ms from all analyses and computed spike counts and peak Ca^2+^ over the steady-state window *t* = 500–3000 ms (i.e., *T*_stim_ = 2.5 s at 80 Hz → *N*_pulses_ = 200).

### Dose-response analysis

Extracellular Mg^2+^ concentration was varied from 0.2 to 2.5 mM. This range spans from severely deficient to moderately elevated levels relative to the physiological concentration of approximately 0.8–1.0 mM in cerebrospinal fluid [24]. High-resolution sampling (0.1 mM steps) was performed in the critical therapeutic region (1.0–2.5 mM) to precisely identify optimal concentrations.

### Intervention timing protocol

To assess the temporal window for neuroprotection, we simulated an excitotoxic stress protocol (80 Hz stimulation from *t* = 0.5 s to *t* = 4.5 s, total 4 s stress duration) with Mg^2+^ supplementation (from 0.2 mM to 1.8 mM) applied at varying delays: pre-treatment, at stress onset (+0 s), and delayed by +0.1, + 0.2, + 0.3, + 0.4, + 0.5, + 0.75, + 1, + 2, and +3 s relative to stress onset. Simulations were run to 6 s to capture post-stress dynamics; peak calcium metrics were evaluated during the defined stress window. This protocol models clinical scenarios where Mg^2+^ therapy might be initiated at different times relative to ischemic onset [22].

## Analysis metrics

### Spike detection

Action potentials were detected using a threshold crossing method (*V* > −20 mV). Spike probability was calculated as the ratio of detected spikes to expected stimulus pulses during the steady-state period. Spike loss was defined as 100×(1−spike probability)%.

### Calcium metrics

Peak intracellular calcium, $[{\text{Ca}}^{2+}{]}_{i,\text{peak}}$, was defined as the maximum $[{\text{Ca}}^{2+}{]}_{i}$ reached during the analysis window (500–3000 ms for dose-response analysis; 500–4500 ms for intervention timing analysis, corresponding to the 0.5–4.5 s stress window). This metric captures the maximum calcium load experienced by the cell, which determines excitotoxic vulnerability.

### Therapeutic criteria

Two criteria defined the therapeutic window, reflecting the dual goals of neuroprotection and function preservation [10]: (1) **Neuroprotection**: Peak $[{\text{Ca}}^{2+}{]}_{i}<1.0\;\mu$M (model toxicity threshold). This threshold is supported by experimental evidence that sustained Ca^2+^ elevations above this level are associated with excitotoxic injury [14,43], and is consistent with thresholds used in established computational models of excitotoxicity [42]. Sensitivity analysis was performed across the range of 0.6–1.4 μM; the therapeutic window was robust for thresholds between 1.0–1.3 μM, and no window exists below 1.0 μM (S2 Fig). (2) **Function preservation**: Spike reduction ≤20% relative to baseline (0.2 mM Mg^2+^). This threshold was chosen based on clinical observations that visual function remains largely preserved with modest RGC loss, while deficits become detectable when loss exceeds ∼30% [5]. The framework allows adjustment of this criterion based on specific clinical priorities. The optimal therapeutic range was defined as Mg^2+^ concentrations satisfying both criteria simultaneously.

### Protection efficacy

For intervention timing analysis, protection efficacy was calculated as:


$$\begin{array}{c} \text{Protection}=\frac{[{\text{Ca}}^{2+}{]}_{\text{peak,no Mg}}-[{\text{Ca}}^{2+}{]}_{\text{peak,intervention}}}{[{\text{Ca}}^{2+}{]}_{\text{peak,no Mg}}-[{\text{Ca}}^{2+}{]}_{\text{peak,pre-treated}}}\times 100\% \end{array}$$ (13)

### Numerical methods

All simulations were performed in MATLAB R2023b (MathWorks, Natick, MA) using the forward Euler method with a time step of *dt* = 0.02 ms. Numerical accuracy was verified by comparing the forward Euler method against a fourth-order Runge-Kutta (RK4) solver, demonstrating <0.2% difference in peak calcium values under identical conditions (S3 Fig). Convergence analysis with decreasing step sizes confirmed that *dt* = 0.02 ms provides sufficient accuracy while maintaining computational efficiency. All simulations are deterministic; repeated runs under identical parameters yield bit-identical results.

## Results

### Model behavior and Mg^2+^-dependent NMDA block

**Fig 1:**
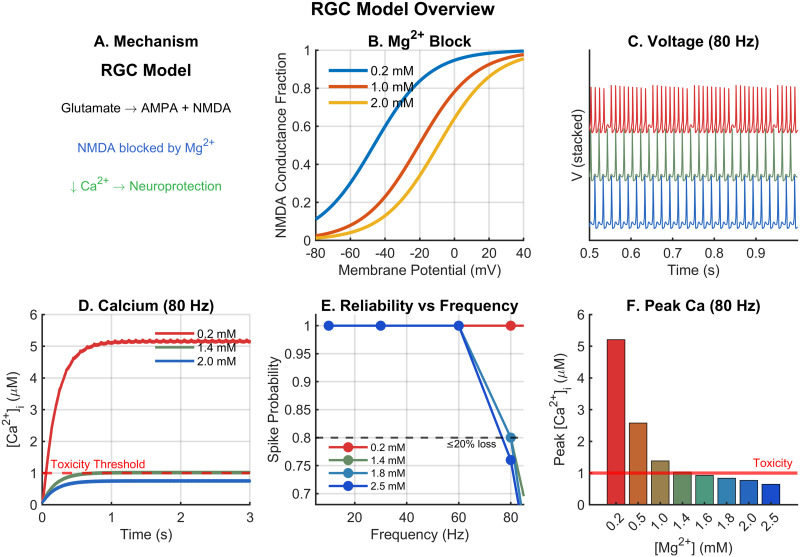
RGC model overview and Mg^2^^+^-dependent dynamics. A: Schematic of the neuroprotection mechanism: glutamate activates AMPA and NMDA receptors; Mg^2+^ blocks NMDA receptors, reducing calcium influx. B: Voltage-dependent Mg^2+^ block of NMDA receptors at three concentrations (0.2, 1.0, 2.0 mM), following the Jahr-Stevens formulation. C: Representative voltage traces at 80 Hz stimulation showing spiking at three Mg^2+^ concentrations. D: Intracellular calcium dynamics at 80 Hz; dashed red line indicates the 1.0 μM toxicity threshold. E: Spike probability across frequencies for all Mg^2+^ concentrations; dashed line at 0.8 indicates 20% spike loss. F: Peak intracellular calcium at 80 Hz for each Mg^2+^ concentration; red line indicates toxicity threshold. The shaded green region indicates concentrations within the therapeutic window.

The RGC model exhibited physiologically realistic spiking behavior in response to glutamatergic stimulation (Fig 1), consistent with experimental recordings from mammalian RGCs [36,37]. The neuroprotection mechanism (Fig 1A) involves glutamate activation of both AMPA and NMDA receptors, with Mg^2+^ selectively blocking NMDA receptors to reduce calcium influx while preserving AMPA-mediated fast transmission. At low extracellular Mg^2+^ (0.2 mM), the neuron reliably followed input stimuli across all tested frequencies, with strong NMDA receptor activation driving substantial calcium influx. The voltage-dependent Mg^2+^ block function (Fig 1B) demonstrated that at resting potential (−65 mV), NMDA conductance was reduced to approximately 3%, 6%, and 24% of maximum at Mg^2+^ concentrations of 2.0, 1.0, and 0.2 mM, respectively, in agreement with experimental measurements [16,18]. Representative voltage traces (Fig 1C) demonstrate maintained spiking across Mg^2+^ concentrations at 80 Hz, though with reduced reliability at higher concentrations.

Increasing extracellular Mg^2+^ progressively reduced intracellular calcium accumulation (Fig 1D, F). At 80 Hz stimulation, peak calcium decreased from 4.59 μM at 0.2 mM Mg^2+^ to 0.84 μM at 2.0 mM Mg^2+^, representing an 82% reduction (Fig 1F). Notably, spike probability remained near 1.0 at frequencies up to 60 Hz regardless of Mg^2+^ concentration, indicating that AMPA receptor-mediated currents plus intrinsic conductances were sufficient to maintain transmission reliability at these frequencies (Fig 1E).

### Frequency-dependent effects of Mg^2+^ on spike output

**Fig 2:**
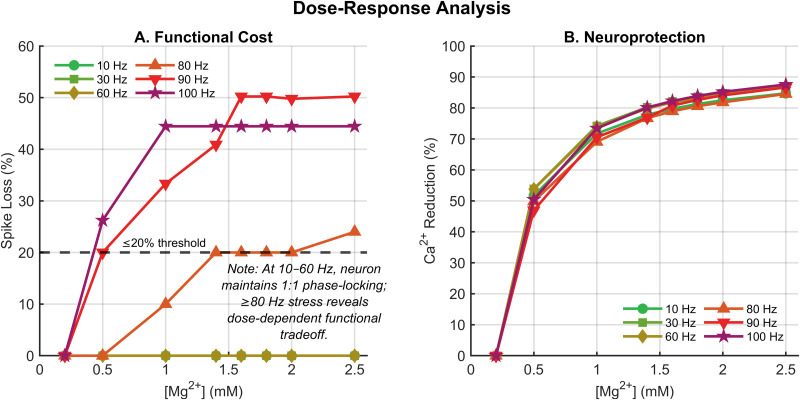
Dose-response analysis across stimulation frequencies. A: Spike loss relative to 0.2 mM baseline as a function of Mg^2+^ concentration. Note that only excitotoxic frequencies (≥80 Hz) produce spike loss, with a plateau at 20% for 1.4–2.0 mM. At 90–100 Hz, spike loss exceeds 20% across all concentrations, closing the therapeutic window. B: Calcium reduction as a function of Mg^2+^ concentration, showing monotonic improvement at all frequencies.

Dose-response analysis revealed a striking frequency-dependent effect of Mg^2+^ on spike output (Fig 2A). At physiological frequencies (10–60 Hz), spike reduction remained at 0% across all Mg^2+^ concentrations tested, demonstrating that NMDA receptor block did not compromise synaptic transmission under normal conditions. In contrast, at excitotoxic frequencies (80 Hz), Mg^2+^ concentrations ≥1.0 mM progressively reduced spike output, with reduction reaching 10% at 1.0 mM and plateauing at 20% for concentrations between 1.4–2.0 mM. It is important to distinguish two related ranges: the spike loss plateau extends across 1.4–2.0 mM, while the optimal therapeutic window—where calcium additionally falls below the 1.0 μM toxicity threshold—is the narrower subset of 1.6–2.0 mM. The 80 Hz frequency represents a critical boundary: at 60 Hz and below, therapeutic windows remain relatively wide (≥1.1 mM), while at frequencies above 80 Hz (90, 100 Hz; Fig 2), no Mg^2+^ concentration simultaneously satisfies both neuroprotection and function criteria—the window closes entirely.

This spike loss plateau represents a critical finding: within the 1.4–2.0 mM range, spike reduction remained constant at 20% while calcium reduction continued to improve (Fig 2B). This dissociation between spike output and calcium dynamics reflects the distinct roles of AMPA and NMDA receptors: AMPA receptors drive fast spiking while NMDA receptors, with their slower kinetics ($\tau_{\text{decay}}=80$ ms), provide temporal summation and calcium entry [41].

### Identification of therapeutic windows

**Fig 3:**
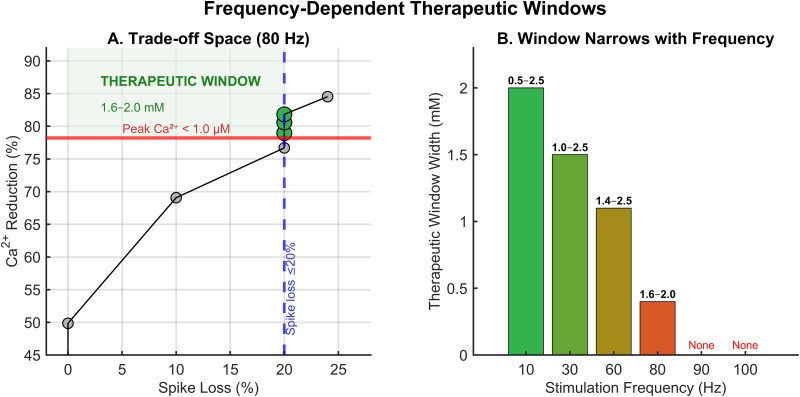
Frequency-dependent therapeutic windows. A: Trade-off space at 80 Hz plotting calcium reduction against spike loss. Green points indicate optimal concentrations satisfying both criteria (calcium < 1.0 μM and spike loss ≤ 20%). The green shaded region indicates the therapeutic window (1.6–2.0 mM, labeled “OPTIMAL”). B: Therapeutic window width decreases with increasing stimulation frequency, from 2.0 mM at 10 Hz to 0.4 mM at 80 Hz. At frequencies >80 Hz, no window exists (“None” label).

The trade-off between neuroprotection and function preservation defined frequency-dependent therapeutic windows (Fig 3). At 80 Hz, plotting calcium reduction against spike reduction revealed an optimal zone where both criteria were satisfied (Fig 3A). Concentrations below 1.6 mM failed to reduce calcium below the toxicity threshold, while concentrations above 2.0 mM exceeded the 20% spike loss limit.

High-resolution analysis (0.1 mM steps) precisely identified the optimal range at 80 Hz as 1.6–2.0 mM (S1 Fig, S1 Table). Within this range, all concentrations achieved peak calcium below 1.0 μM (ranging from 0.97 μM at 1.6 mM to 0.84 μM at 2.0 mM) while maintaining spike loss at exactly 20% (160 of 200 expected spikes). This narrow therapeutic window corresponds to mild hypermagnesemia, a clinically achievable target [24]. Sensitivity analysis confirmed robustness across the tested threshold range, with the therapeutic window maintained for Ca^2+^ toxicity thresholds between 1.0–1.3 μM (S2 Fig).

The therapeutic window width decreased with increasing stimulation frequency (Fig 3B): 10 Hz: 0.5–2.5 mM (width: 2.0 mM); 30 Hz: 1.0–2.5 mM (width: 1.5 mM); 60 Hz: 1.4–2.5 mM (width: 1.1 mM); 80 Hz: 1.6–2.0 mM (width: 0.4 mM); 90 Hz and 100 Hz: no therapeutic window exists (spike loss exceeds 20% at all neuroprotective concentrations). This narrowing reflects the increasing reliance on NMDA receptor-mediated currents for maintaining spike output at high frequencies [25].

### Temporal constraints on Mg^2+^ intervention

**Fig 4:**
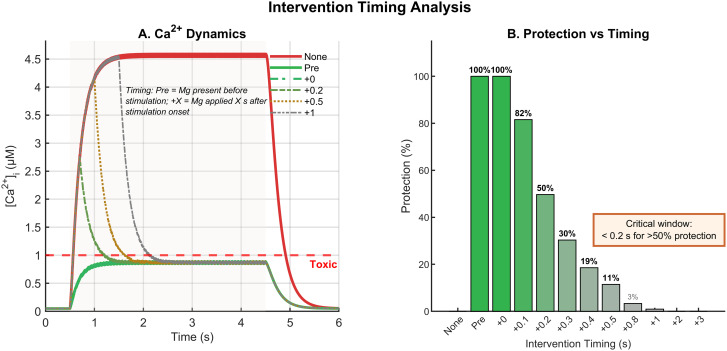
Intervention timing determines protection efficacy. A: Calcium dynamics during 80 Hz stress (0.5–4.5 s) with Mg^2+^ supplementation at different delays. Pre-treatment and +0 s intervention maintain calcium near the toxicity threshold, while delayed interventions fail to prevent calcium overload. B: Protection efficacy as a function of intervention timing. Pre-treatment and immediate intervention achieve 100% protection; efficacy drops to 11% at +0.5 s delay and approaches zero for delays ≥ 1 s. The critical window for ≥50% protection is ≤0.2 s (indicated by shaded region); this reflects the rapid Ca^2+^ rise under our abrupt-onset protocol rather than a literal clinical deadline.

The intervention timing protocol demonstrated that Mg^2+^ protection efficacy is highly sensitive to intervention delay (Fig 4). Pre-treatment and intervention at stress onset (+0 s) both achieved 100% protection efficacy, maintaining peak calcium near 1.0 μM. However, protection declined steeply with delay: ≥50% protection required intervention within 0.2 s (efficacy at +0.1 s: 82%; at +0.2 s: 50%), while by +0.5 s delay efficacy dropped to approximately 11%, and delays of ≥1 s provided negligible protection (≤3%; Fig 4B).

The calcium dynamics underlying this temporal sensitivity are evident in Fig 4A. Without Mg^2+^ supplementation, calcium rose rapidly during stress, reaching toxic levels within the first second. Delayed interventions failed to prevent this initial calcium surge, and although Mg^2+^ application eventually reduced the rate of calcium accumulation, peak levels had already exceeded the toxicity threshold. These findings parallel clinical observations that early intervention is critical for neuroprotective efficacy in stroke and other ischemic conditions [22,23].

**Fig 5:**
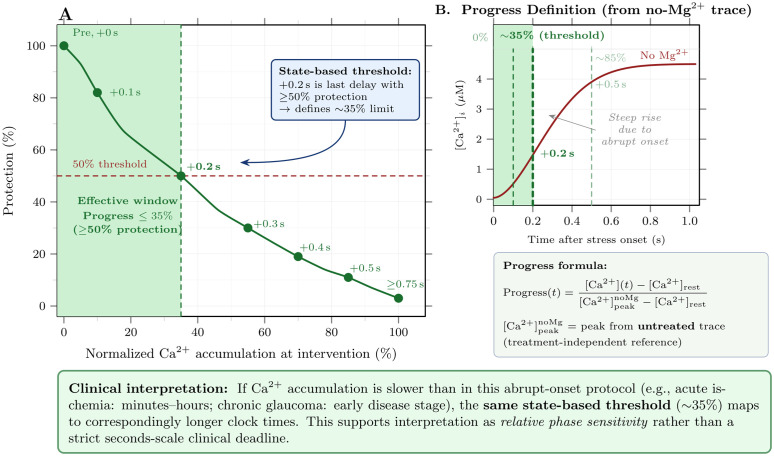
Protection efficacy depends on Ca^2^^+^ progress, not absolute time. A: Protection efficacy replotted against normalized Ca^2+^ accumulation at the moment of Mg^2+^ application. Efficacy at each delay is computed using Eq. 13: the fraction of peak calcium reduction achieved relative to the pre-treated condition (100% = pre-treatment performance; 0% = no intervention). Ca^2+^ progress is defined as $\left([{Ca}^{2+}](t)-[{Ca}^{2+}{]}_{rest})\;/\;([{Ca}^{2+}{]}_{peak}^{noMg}-[{Ca}^{2+}{]}_{rest}\right)$, where $[{Ca}^{2+}{]}_{peak}^{noMg}$ is the peak from the *untreated* trace—a treatment-independent reference for excitotoxic phase. The critical state-based threshold for ≥50% protection (∼35% Ca^2+^ progress; dashed green line) is derived from the + 0.2 s condition, the last delay yielding ≥50% protection. B: Derivation of progress values from the no-Mg^2+^ Ca^2+^ trace. The steep rise reflects the abrupt-onset protocol used in simulations. *Because Ca*^*2+*^ *accumulation in vivo is typically slower than in this protocol (acute ischemia: minutes–hours; chronic glaucoma: early disease stage), the same state-based threshold maps to correspondingly longer clock times. The model thus predicts relative phase sensitivity—intervention efficacy depends on when during the Ca*^*2+*^ *rise treatment occurs—rather than a strict seconds-scale clinical deadline.*

Because excitotoxic drive begins abruptly in our protocol, the seconds-scale intervention window reflects the rapid early rise of $[{\text{Ca}}^{2+}{]}_{i}$ rather than a fundamental biological constraint. We therefore re-expressed timing sensitivity in terms of normalized Ca^2+^ progress—the fraction of untreated peak accumulation reached at the moment of intervention (Fig 5A). Under this state-based representation, ≥50% protection requires intervention before ∼35% of the untreated Ca^2+^ peak is reached, regardless of the absolute time required. The derivation of progress values from the no-Mg^2+^ Ca^2+^ trace is shown in Fig 5B, which illustrates the steep rise characteristic of the abrupt-onset protocol used in simulations.

### Mechanistic basis of Mg^2+^ neuroprotection

**Fig 6:**
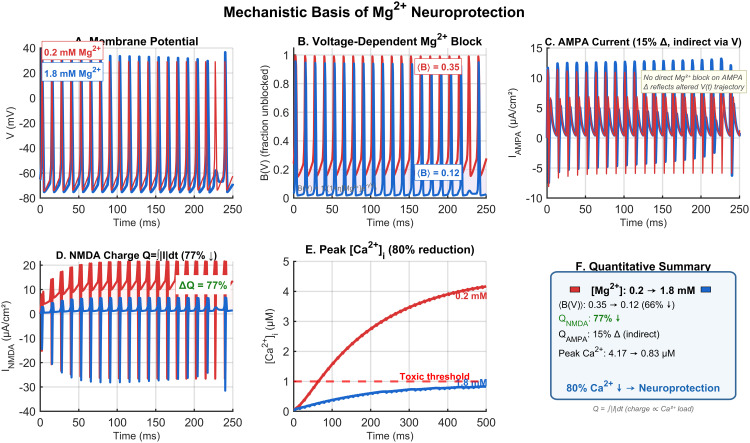
Mechanistic basis of Mg^2^^+^ neuroprotection. A: Membrane potential at 80 Hz comparing low (0.2 mM, red) and therapeutic (1.8 mM, blue) Mg^2+^. B: Voltage-dependent Mg^2+^ factor *B*(*V*) (fraction unblocked; lower values indicate stronger block), with mean ⟨B⟩ decreasing from 0.35 (0.2 mM) to 0.12 (1.8 mM). C: AMPA current (no direct Mg^2+^ block; the ∼15% change reflects the altered *V*(*t*) trajectory indirectly). D: NMDA current and integrated charge $Q_{NMDA}=\int |I_{NMDA}|\;dt$, showing a 77% reduction a*t* 1.8 mM. E: Intracellular calcium accumulation and peak $[{Ca}^{2+}{]}_{i}$ within the displayed 250 ms window (4.17 → 0.83 μM; 80% reduction); note that the full-protocol peak at 0.2 mM Mg^2+^ over the complete 2500 ms analysis window is 4.59 μM, as reported in the Results. Dashed line indicates the toxicity threshold. F: Quantitative summary of the neuroprotection cascade linking Mg^2+^ elevation to reduced NMDA-driven Ca^2+^ load.

Detailed analysis of synaptic currents confirmed the mechanistic cascade underlying Mg^2+^ neuroprotection (Fig 6). Comparison of low (0.2 mM) versus therapeutic (1.8 mM) Mg^2+^ at 80 Hz revealed that elevated Mg^2+^ substantially reduced NMDA current amplitude (Fig 6B), with peak NMDA current amplitude decreasing by approximately 85% (integrated charge *Q*_NMDA_ reduced by 77%; Fig 6D). Despite the strong NMDA block, AMPA current magnitude changed only modestly (∼15%) due to the indirectly altered voltage trajectory (Fig 6C), confirming that the Mg^2+^ block acts selectively on NMDA receptors without directly suppressing AMPA-mediated transmission. This reduction in NMDA current directly translated to reduced calcium influx (Fig 6E), with peak calcium remaining below the 1.0 μM threshold throughout stimulation at 1.8 mM Mg^2+^, compared to exceeding 4 μM at 0.2 mM Mg^2+^.

Importantly, despite the dramatic reduction in NMDA current, spike generation was only modestly affected (Fig 6A). Both conditions produced regular spiking, though with slightly reduced amplitude and reliability at elevated Mg^2+^. This preservation of function reflects the role of AMPA receptors and intrinsic conductances in maintaining spike generation [11]. The mechanistic cascade is summarized schematically (Fig 6F): increased [Mg^2+^]_ext_ → enhanced NMDA block → reduced *I*_NMDA_ → decreased Ca^2+^ influx → neuroprotection.

## Discussion

This study presents a comprehensive computational investigation of Mg^2+^-mediated neuroprotection in RGCs during glutamatergic excitotoxicity. Our biophysically detailed model reveals several key findings with important implications for understanding and potentially treating conditions involving RGC degeneration, including glaucoma and retinal ischemia.

### Summary of principal findings

Our simulations demonstrate that extracellular Mg^2+^ provides frequency-dependent neuroprotection through voltage-dependent NMDA receptor block. At physiological stimulation frequencies (10–60 Hz), elevated Mg^2+^ substantially reduced intracellular calcium accumulation (50–85%, across the 1.0–2.5 mM range) without compromising spike output, indicating that AMPA receptor-mediated transmission alone is sufficient to maintain synaptic function under normal conditions.

However, at the modeled excitotoxic boundary (80 Hz), a narrow therapeutic window of 1.6–2.0 mM emerged, where both neuroprotection (peak Ca^2+^ below 1.0 μM) and function preservation (spike loss ≤20%) criteria were satisfied simultaneously. This finding is clinically relevant, as concentrations in this range represent mild hypermagnesemia that is achievable through therapeutic intervention [22,24].

The spike loss plateau phenomenon observed at 80 Hz represents a particularly important finding. Within the 1.4–2.0 mM range, spike reduction remained constant at 20% while calcium accumulation continued to decrease. However, it is the narrower 1.6–2.0 mM subset—where calcium additionally falls below the 1.0 μM toxicity threshold—that constitutes the true therapeutic window, creating an “optimal zone” for simultaneous neuroprotection and function preservation. This dissociation reflects the distinct biophysical roles of AMPA and NMDA receptors [11,41].

### Comparison with previous computational studies

Our modeling approach extends previous computational investigations of glutamatergic synaptic transmission and NMDA receptor dynamics. The synaptic kinetics and NMDA receptor formulations employed in our model are consistent with established frameworks [31,38]. The voltage-dependent Mg^2+^ block formulation follows the Jahr-Stevens model [18], which has been extensively validated against experimental data [11,25].

Previous computational work on opioid-induced synaptic plasticity has demonstrated that modifications to NMDA receptor function can profoundly influence calcium dynamics and downstream signaling cascades [12,31]. Interestingly, computational studies of cocaine’s effects on neuronal excitability have shown that drugs affecting potassium conductances can dramatically alter action potential generation [46]. While our model focuses on Mg^2+^ modulation of NMDA receptors, both studies highlight how relatively modest changes in ionic conductances can produce qualitatively different firing patterns.

### Mechanistic insights into Mg^2+^ neuroprotection

The mechanistic cascade underlying Mg^2+^ neuroprotection in our model is consistent with the established physiology of NMDA receptor-mediated excitotoxicity [14,16,17]. Recent cryo-EM structural studies have provided unprecedented insights into the molecular basis of Mg^2+^ action on NMDA receptors, identifying three distinct binding sites [19]. The approximately 85% reduction in NMDA current amplitude observed at therapeutic Mg^2+^ concentrations directly translates to reduced calcium loading.

A critical insight from our model is that the voltage-dependence of the Mg^2+^ block creates a natural activity-dependent filter. This mechanism differs fundamentally from competitive NMDA receptor antagonists, which reduce receptor function in a voltage-independent manner [15]. The voltage-dependent nature of Mg^2+^ block may explain why Mg^2+^ supplementation has shown promise in neuroprotection without adverse cognitive effects [22].

### Biological interpretation of intervention timing

The steep decline in protection with intervention delay (≥50% protection requires ≤0.2 s; ∼11% by 0.5 s in our protocol) should not be interpreted as a literal clinical deadline. Rather, it reflects the importance of *relative timing* within the excitotoxic process. In our idealized protocol, stress begins abruptly at full intensity; clinical conditions such as retinal ischemia or glaucoma involve gradual glutamate accumulation over minutes to hours. The same principle holds: earlier intervention, before the cell has accumulated substantial Ca^2+^ load, yields greater protection—but the absolute timescale is set by the rate of Ca^2+^ accumulation in the specific pathological context, not by the seconds-scale protocol used here.

More directly, our results provide strong support for *prophylactic* Mg^2+^ supplementation in at-risk scenarios (e.g., patients undergoing ocular surgery, those with acute angle-closure glaucoma attacks, or retinal vascular occlusion), where treatment can be initiated before or concurrent with excitotoxic stress onset. The 100% protection efficacy observed with pre-treatment underscores this preventive strategy. For slowly progressive conditions such as glaucoma, the timing results suggest that neuroprotective intervention early in the disease course—before cumulative calcium-mediated damage becomes extensive—offers the greatest potential benefit.

Our quantitative reanalysis (Fig 5) provides a rigorous framework for this interpretation. By re-expressing timing in terms of normalized Ca^2+^ progress—the fraction of untreated peak accumulation at intervention—we demonstrate that protection efficacy is determined by the *state of the cell* (Ca^2+^ load) rather than absolute clock time. The critical state-based threshold for ≥50% protection corresponds to ∼35% Ca^2+^ progress. In biological settings where excitotoxicity develops more gradually than in our abrupt-onset simulations, the same Ca^2+^-state threshold would correspond to longer absolute intervention windows. This state-based interpretation reconciles our seconds-scale simulation results with the clinical reality that neuroprotective interventions in stroke show diminishing returns with treatment delay [22,23], while avoiding the implication of an unrealistic seconds-scale clinical deadline.

### Validation against experimental and clinical data

Our predicted optimal Mg^2+^ range (1.6–2.0 mM) corresponds to mild hypermagnesemia that is readily achievable clinically. Normal serum Mg^2+^ is 0.7–1.0 mM, while therapeutic hypermagnesemia protocols (e.g., for eclampsia or neuroprotection trials) routinely achieve 2.0–3.5 mM [22]. The FAST-MAG trial demonstrated that serum levels of 2.5–3.0 mM can be achieved within 2 hours of prehospital administration [23].

Several experimental findings align with our predictions. Studies of retinal ischemia have reported reduced RGC loss with Mg^2+^ supplementation at concentrations within our therapeutic range [3]. Conversely, the Intravenous Magnesium Efficacy in Stroke (IMAGES) trial found no benefit from Mg^2+^ in stroke when administered late (median 7 hours post-onset) [47], consistent with our finding that delayed intervention provides negligible protection. This framework may help explain mixed clinical results: efficacy depends critically on both concentration (within the narrow therapeutic window) and timing (early intervention).

### Translational considerations for Mg^2+^ concentrations

Retinal extracellular Mg^2+^ is regulated by the blood-retinal barrier, with concentrations typically 10–15% below serum levels (similar to cerebrospinal fluid). Achieving our therapeutic range of 1.6–2.0 mM in retinal tissue would therefore require serum levels of approximately 1.8–2.3 mM—within the range routinely achieved in clinical hypermagnesemia protocols [22,23]. Local delivery routes such as intravitreal injection could potentially achieve therapeutic retinal concentrations with lower systemic exposure, though pharmacokinetic studies specific to retinal Mg^2+^ distribution would be needed to optimize such approaches.

### Understanding mixed clinical results

Several clinical trials of Mg^2+^ neuroprotection have yielded disappointing results, which our computational framework may help contextualize. The IMAGES trial found no benefit from intravenous Mg^2+^ in stroke, but treatment was initiated late (median 7 hours post-symptom onset) and achieved serum levels (1.5–1.8 mM) at the lower end of our therapeutic range [47]. Our model predicts negligible protection with delayed intervention (≥1 s in model time) and suggests that concentrations below 1.6 mM may provide insufficient NMDA block at excitotoxic frequencies.

Similarly, the FAST-MAG trial, despite achieving higher serum Mg^2+^ levels with early prehospital administration, showed no overall benefit—though subgroup analyses hinted at efficacy in specific stroke subtypes [23]. Our findings suggest that population heterogeneity in excitotoxic severity (analogous to different stimulation frequencies) could produce variable responses, as the therapeutic window narrows dramatically at higher stress levels. This hypothesis remains to be tested experimentally.

These considerations highlight the potential value of computational modeling: by identifying critical dependencies on concentration and timing, such frameworks may help guide trial design toward parameter regimes with higher probability of success.

### Implications for RGC neuroprotection

Glaucoma, affecting over 70 million people worldwide, is characterized by progressive RGC loss [4,5]. Recent studies have identified caspase-mediated pathways as promising therapeutic targets [6], and emerging evidence supports synergistic neuroprotective approaches [9]. Additionally, neurotrophic factor delivery through gene therapy vectors has shown remarkable promise [48]. Our findings suggest that Mg^2+^ supplementation in the range of 1.6–2.0 mM could provide meaningful neuroprotection and may act synergistically with these emerging strategies.

### Model limitations and future directions

We acknowledge several limitations inherent to our computational approach. Our RGC model uses a single-compartment approach that cannot capture dendritic Ca^2+^ microdomains [49]. Dendritic compartmentalization of NMDA receptors could create localized Ca^2+^ microdomains with peak concentrations significantly exceeding our soma-averaged estimates. This spatial heterogeneity has two implications: (1) local Ca^2+^ might reach toxic levels more rapidly, potentially increasing timing sensitivity beyond our predictions, and (2) spatial isolation could provide compartmentalized protection, possibly enabling efficacy at lower bulk Mg^2+^ concentrations. Our single-compartment estimates should therefore be considered conservative, representing an upper bound on the required therapeutic concentration. Future multi-compartment models incorporating realistic RGC dendritic morphology and NMDA receptor distributions would help quantify these spatial effects.

Glutamate input was modeled as periodic pulses; real excitotoxic environments involve irregular patterns. The model omits glial glutamate clearance and network-level interactions [31]. The criteria defining our therapeutic window are physiologically motivated but simplified [14]. The steep decline in protection with delay (≥50% protection requires ≤0.2 s) arises from our specific abrupt-onset protocol; clinical ischemia develops more gradually, and the equivalent timescale would be correspondingly longer. The Mg^2+^ concentrations cannot be directly equated to clinical dosing [24]. Finally, these predictions require experimental validation.

Despite these limitations, the model provides a mechanistically grounded framework, identifies testable predictions, and establishes a computational platform that can be extended as experimental data become available.

## Conclusions

This computational study provides systematic, quantitative analysis of Mg^2+^-mediated neuroprotection in RGCs during excitotoxicity. Key findings include: (1) frequency-dependent therapeutic windows that narrow from 2.0 mM width at 10 Hz to 0.4 mM at 80 Hz, with complete window closure at 90–100 Hz; (2) optimal range of 1.6–2.0 mM at the modeled excitotoxic boundary (80 Hz), corresponding to the narrower therapeutic subset within the 1.4–2.0 mM spike-loss plateau; (3) spike loss plateau creating a “sweet spot” for neuroprotection; (4) steep decline in protection efficacy with intervention delay (≥50% protection requires ≤0.2 s in our abrupt-onset protocol, reflecting relative Ca^2+^-phase sensitivity rather than a literal clinical deadline); and (5) mechanistic basis through enhanced NMDA block reducing calcium influx while ‌‌preserving AMPA-mediated spiking.

These findings provide quantitative guidance for Mg^2+^-based neuroprotective strategies in glaucoma and retinal ischemia. The framework may help explain previously mixed clinical results by identifying critical dependencies on concentration and timing, and supports consideration of prophylactic strategies in at-risk patients. Future experimental validation will be essential for clinical translation.

## Supporting information

S1 Fig High-resolution therapeutic window analysis at 80 Hz. A: Dose-response with 0.1 mM resolution showing spike loss and peak calcium. B: Trade-off space visualization identifying the 1.6–2.0 mM range as optimal, with color-coded classification of each concentration.
(TIF)

S2 FigCa^2+^ toxicity threshold sensitivity analysis.A: Therapeutic window width as a function of toxicity threshold. B: Optimal Mg^2+^ range boundaries across threshold values. C: Dose-response curve with default threshold (1.0 μM) highlighted. The therapeutic window exists for toxicity thresholds ≥1.0 μM and remains robust between 1.0–1.3 μM, confirming robustness of findings.(TIF)

S3 FigNumerical method validation.A: Convergence analysis showing peak $[{\text{Ca}}^{2+}{]}_{i}$ vs. time step for the Forward Euler method, with RK4 reference. B: Relative error vs. reference solution (Euler dt=5 μs). C: Validation summary confirming <0.2% error at dt=20 μs.(TIF)

S1 TableHigh-resolution simulation results at 80 Hz.Spike counts, spike loss percentage, and peak intracellular calcium for each Mg^2+^ concentration tested. Protocol: *T*_stim_ = 2.5 s, *f* = 80 Hz $\to N_{\text{pulses}}=200$; Spike Loss $=100\times (1-N_{\text{spikes}}/N_{\text{pulses}})$. Concentrations satisfying both therapeutic criteria (peak $[{\text{Ca}}^{2+}{]}_{i}<1.0\;\mu$M *and* spike loss ≤20%) are marked with * and highlighted.(PDF)

S2 Table Complete model parameters. Complete parameter values with units, descriptions, and primary references for all intrinsic conductances, synaptic kinetics, Mg^2+^ block, calcium dynamics, and therapeutic criteria used in the computational model.
(PDF)
