# Supplementary figures and images for: Magnesium neuroprotection in retinal ganglion cells: A computational study of frequency-dependent therapeutic windows and intervention timing
Source: PLoS One. 2026 Jun 1;21(6):e0348068. doi: 10.1371/journal.pone.0348068 (PMC13225435; doi:10.1371/journal.pone.0348068)

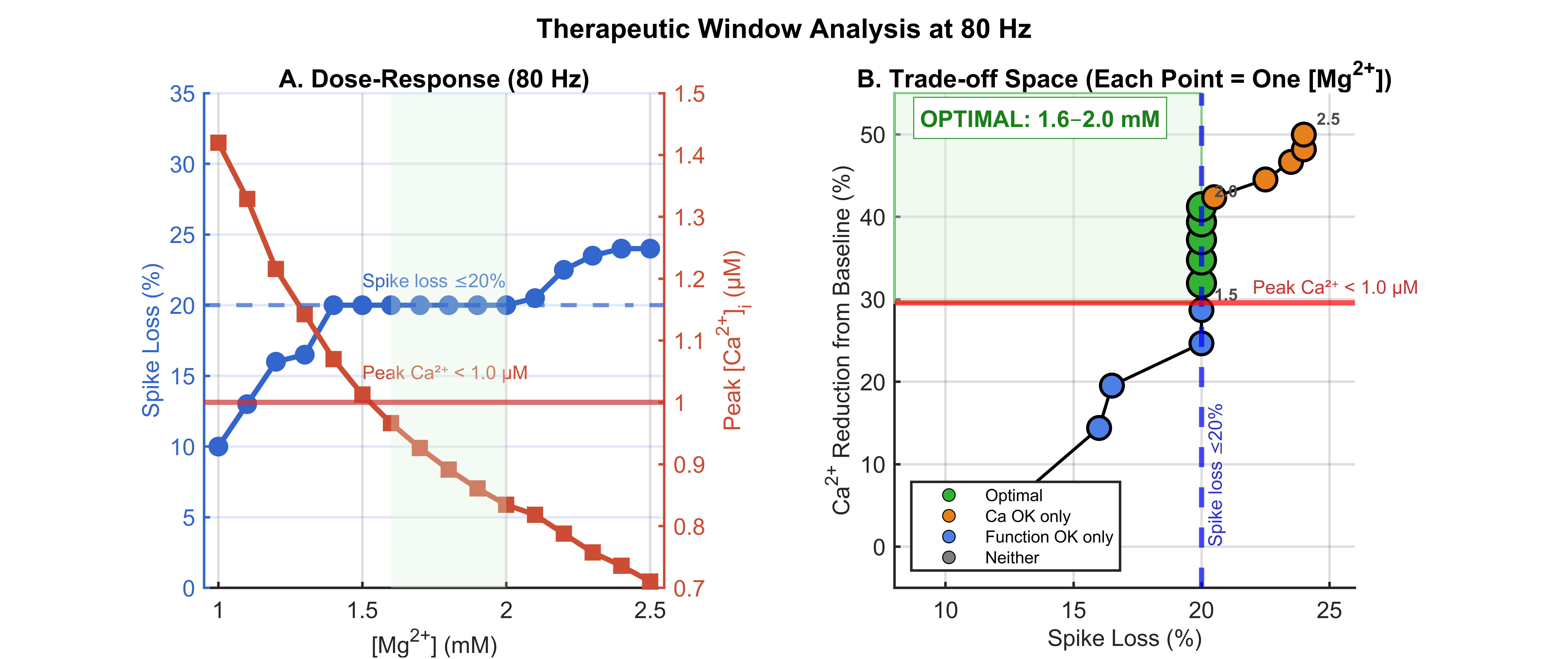

Supplement: S1 Fig — A: Dose-response with 0.1 mM resolution showing spike loss and peak calcium. B: Trade-off space visualization identifying the 1.6–2.0 mM range as optimal, with color-coded classification of each concentration. (TIF) [file pone.0348068.s001.tif]

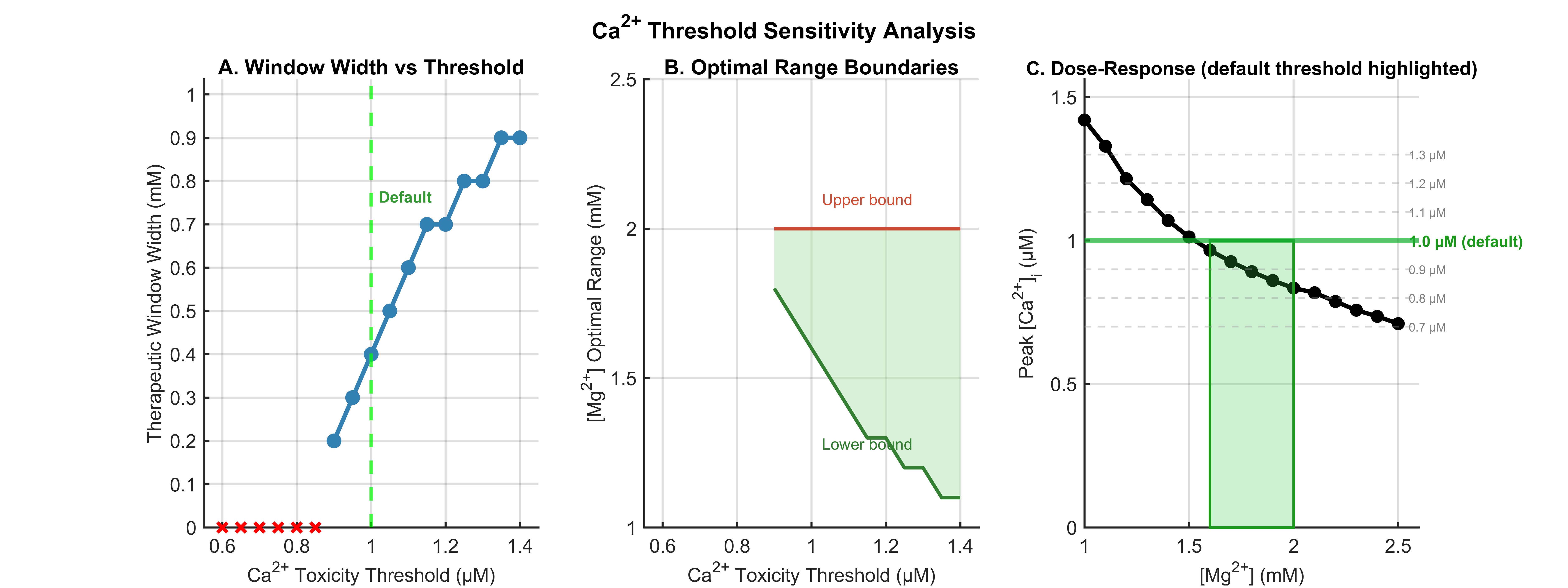

Supplement: S2 Fig — A: Therapeutic window width as a function of toxicity threshold. B: Optimal Mg2+ range boundaries across threshold values. C: Dose-response curve with default threshold (1.0 μM) highlighted. The therapeutic window exists for toxicity thresholds ≥1.0 μM and remains robust between 1.0–1.3 μM, confirming robustness of findings. (TIF) [file pone.0348068.s002.tif]

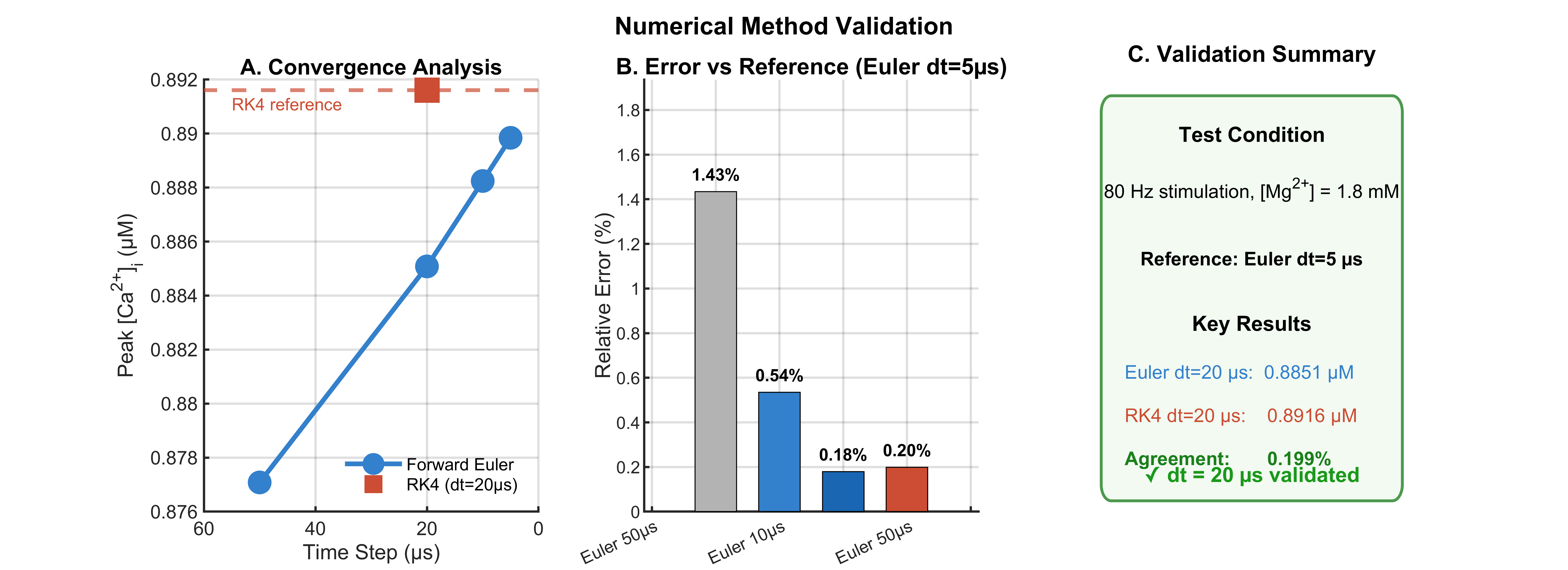

Supplement: S3 Fig — A: Convergence analysis showing peak [Ca2+]i vs. time step for the Forward Euler method, with RK4 reference. B: Relative error vs. reference solution (Euler dt=5 μs). C: Validation summary confirming <0.2% error at dt=20 μs. (TIF) [file pone.0348068.s003.tif]
