# Supplementary material for: Magnesium neuroprotection in retinal ganglion cells: A computational study of frequency-dependent therapeutic windows and intervention timing
Source: PLoS One. 2026 Jun 1;21(6):e0348068. doi: 10.1371/journal.pone.0348068 (PMC13225435; doi:10.1371/journal.pone.0348068)
Supplement: S1 Table — Spike counts, spike loss percentage, and peak intracellular calcium for each Mg2+ concentration tested. Protocol: Tstim = 2.5 s, f = 80 Hz →Npulses=200; Spike Loss =100×(1−Nspikes/Npulses). Concentrations satisfying both therapeutic criteria (peak [Ca2+]i<1.0 μM and spike loss ≤20%) are marked with * and highlighted. (PDF) [file pone.0348068.s004.pdf]

S1 Table. High-resolution simulation results at 80 Hz.

Spike counts, spike loss percentage, and peak intracellular calcium for each Mg^{2+} concentration tested. Protocol: T_{stim} = 2.5 s, f = 80 Hz → N_{pulses} = 200; Spike Loss = 100 × (1 − N_{spikes}/N_{pulses}). Concentrations satisfying both therapeutic criteria (peak $[Ca^{2+}]_i$ < 1.0 μM *and* spike loss ≤ 20%) are marked with * and highlighted.

$[Mg^{2+}]$	Spikes	Loss (%)	Peak Ca (μM)	Ca < 1.0?	Optimal
1.0 mM	180/200	10.0	1.420	No	
1.1 mM	174/200	13.0	1.329	No	
1.2 mM	168/200	16.0	1.216	No	
1.3 mM	167/200	16.5	1.143	No	
1.4 mM	160/200	20.0	1.070	No	
1.5 mM	160/200	20.0	1.013	No	
1.6 mM	160/200	20.0	0.966	Yes	*
1.7 mM	160/200	20.0	0.926	Yes	*
1.8 mM	160/200	20.0	0.891	Yes	*
1.9 mM	160/200	20.0	0.861	Yes	*
2.0 mM	160/200	20.0	0.835	Yes	*
2.1 mM	159/200	20.5	0.818	Yes	
2.2 mM	155/200	22.5	0.788	Yes	
2.3 mM	153/200	23.5	0.757	Yes	
2.4 mM	152/200	24.0	0.736	Yes	
2.5 mM	152/200	24.0	0.710	Yes	

* Meets both criteria: peak $[Ca^{2+}]_i$ < 1.0 μM and spike loss ≤ 20%.
Optimal therapeutic range: 1.6–2.0 mM.
