# Supplementary material for: Magnesium neuroprotection in retinal ganglion cells: A computational study of frequency-dependent therapeutic windows and intervention timing
Source: PLoS One. 2026 Jun 1;21(6):e0348068. doi: 10.1371/journal.pone.0348068 (PMC13225435; doi:10.1371/journal.pone.0348068)
Supplement: S2 Table — Complete parameter values with units, descriptions, and primary references for all intrinsic conductances, synaptic kinetics, Mg2+ block, calcium dynamics, and therapeutic criteria used in the computational model. (PDF) [file pone.0348068.s005.pdf]

S2 Table. Complete model parameters.

Symbol	Value	Unit	Description	Ref.
Membrane Properties				
C_m	1.0	$\mu F/cm^2$	Membrane capacitance	[1]
V_{rest}	−65	mV	Resting potential	[1]
Reversal Potentials				
E_{Na}	50	mV	Sodium reversal	[1]
E_K	−77	mV	Potassium reversal	[1]
E_L	−54.4	mV	Leak reversal	[1]
E_{Ca}	120	mV	Calcium reversal	[2]
E_{exc}	0	mV	Excitatory synaptic reversal	[3]
Maximal Conductances				
g_{Na}	120	mS/cm^2	Sodium	[1]
g_{Kdr}	36	mS/cm^2	Delayed rectifier K^+	[1]
g_{KA}	8	mS/cm^2	A-type K^+	[4]
g_{CaL}	0.3	mS/cm^2	L-type Ca^{2+}	[2]
g_{KCa}	0.3	mS/cm^2	Ca^{2+}-activated K^+	[4]
g_L	0.35	mS/cm^2	Leak	[1]
g_{AMPA}	0.25	mS/cm^2	AMPA receptor	[3]
g_{NMDA}	1.2	mS/cm^2	NMDA receptor	[3]
Synaptic Kinetics				
$EC_{50,AMPA}$	0.5	mM	AMPA glutamate affinity	[5]
$EC_{50,NMDA}$	2	μM	NMDA glutamate affinity	[6]
$\tau_{AMPA,rise}$	0.3	ms	AMPA rise time	[3]
$\tau_{AMPA,decay}$	3.0	ms	AMPA decay time	[3]
$\tau_{NMDA,rise}$	5.0	ms	NMDA rise time	[7]
$\tau_{NMDA,decay}$	80	ms	NMDA decay time	[7]
Mg^2■ Block (Jahr–Stevens)				
η	0.28	mM^{-1}	Mg^{2+} sensitivity	[8]
γ	0.062	mV^{-1}	Voltage sensitivity	[8]
Calcium Dynamics				
f_{Ca}	0.15	—	Fractional Ca^{2+} current (NMDA)	[9]
$[Ca^{2+}]_{rest}$	0.05	μM	Resting intracellular Ca^{2+}	[10]
τ_{Ca}	200	ms	Ca^{2+} decay time constant	[10]
$k_{Ca,NMDA}$	0.012	—	NMDA→Ca^{2+} scaling factor	Fitted

Symbol	Value	Unit	Description	Ref.
$k_{Ca,CaL}$	0.003	—	L-type→Ca^{2+} scaling factor	Fitted
$K_{d,KCa}$	0.5	μM	KCa half-activation	[4]
n_{KCa}	2	—	KCa Hill coefficient	[4]
Therapeutic Criteria				
$[Ca^{2+}]_{toxic}$	1.0	μM	Toxicity threshold	[11]
Max. spike loss	20	%	Functional preservation limit	Clinical

References in this table use an independent numbering scheme. [1] Hodgkin & Huxley, 1952; [2] Fox et al., 1987; [3] Destexhe et al., 1994; [4] Connor & Stevens, 1971; Sah, 1996; [5] Patneau & Mayer, 1990; [6] Bhalla et al., 1999; [7] Lester et al., 1990; [8] Jahr & Stevens, 1990; [9] Schneggenburger et al., 1993; [10] Clapham, 2007; Bhalla & Iyengar, 1999; [11] Orrenius et al., 2003; Goldberg, 1997.
